# Big data and development sociology: An overview and application on governance and accountability through digitalization in Tanzania

**DOI:** 10.3389/fsoc.2022.909458

**Published:** 2022-11-17

**Authors:** Nicole Schwitter, Alexia Pretari, William Marwa, Simone Lombardini, Ulf Liebe

**Affiliations:** ^1^Department of Sociology, University of Warwick, Coventry, United Kingdom; ^2^Oxfam GB, Oxford, United Kingdom; ^3^Oxfam International, Dar es Salaam, Tanzania

**Keywords:** accountability, big data, development sociology, difference-in-difference, digital data, Tanzania, Twitter

## Abstract

The digital revolution and the widespread use of the internet have changed many realms of empirical social science research. In this paper, we discuss the use of big data in the context of development sociology and highlight its potential as a new source of data. We provide a brief overview of big data and development research, discuss different data types, and review example studies, before introducing our case study on active citizenship in Tanzania which expands on an Oxfam-led impact evaluation. The project aimed at improving community-driven governance and accountability through the use of digital technology. Twitter and other social media platforms were introduced to community animators as a tool to hold national and regional key stakeholders accountable. We retrieve the complete Twitter timelines up to October 2021 from all ~200 community animators and influencers involved in the project (over 1.5 million tweets). We find that animators have started to use Twitter as part of the project, but most have stopped tweeting in the long term. Employing a dynamic difference-in-differences design, we also do not find effects of Oxfam-led training workshops on different aspects of animators' tweeting behavior. While most animators have stopped using Twitter in the long run, a few have continued to use social media to raise local issues and to be part of conversations to this day. Our case study showcases how (big) social media data can be part of an intervention, and we end with recommendations on how to use digital data in development sociology.

## Introduction

The digital revolution and the widespread use of the internet have influenced and changed many realms of empirical social science research. The usages of (big) digital data are flourishing in the growing field of computational social sciences, and novel digital sources of data are becoming popular to gain new insights into old and new questions of the social sciences (Lazer et al., 2009, 2020; Keuschnigg et al., 2017; Salganik, 2018; Edelmann et al., 2020).

Many studies have made use of digital technologies to access rich data sources. Particularly, digital trace data—records of activity undertaken through an online information system such as websites, social media platforms, smartphone apps, or other digital trackers and sensors (Howison et al., 2011; Stier et al., 2019)—are increasingly used as a substitute of or complement to more traditional data sources as their availability tends to allow the time- and cost-effective real-time collection of large amounts of data. While big data come with their traps and biases (Lazer et al., 2014), particularly around representativeness due to access to the internet and/or devices, which are not neutral to race, class, gender, and geography, they can provide a uniquely unobtrusive way to access information from people who are in positions of marginalization and may be reluctant to engage with institutions and institutional players, such as researchers.

An increasing interest in development sociology has emerged throughout the last decades and more and more sociologists work (again) on sociological issues in the context of so-called “developing countries” to reflect on the “development sector” (Viterna and Robertson, 2015). Next to theoretical accounts, empirical research is an integral part of development research. It is therefore natural to consider big data analysis a promising tool in research and intervention studies. In fact, the development sector already explores the possibilities of big data, discussing both the advantages and disadvantages of using various data sources such as satellite images, social media data, or large text corpora for research and evaluation (e.g., Abreu Lopes et al., 2018; Data2X, 2019; York and Bamberger, 2020). Against this background, we aim to shed more light on the nexus between big data and development sociology based on a transdisciplinary collaboration between sociologists and Oxfam^1^. We want to highlight opportunities for analysis with digitally available data in the context of development sociology. To support our argument, we present the collaborative project as a case study and particularly focus on the long-term sustainability analyzable with (big) digital trace data.

This paper is structured as follows: In the next section, we provide a brief overview of the use of big data in development research and discuss different data types. We review example studies and highlight the sociological potential of big data in this context. After this overview, we discuss our own case study on active citizenship in Tanzania where we have used digital trace data from Twitter. We will introduce the study, contextualize it, and discuss important concepts employed. We will present our data and methods, as well as the findings regarding the Twitter activity. We analyze short- and long-term effects of the intervention and assess to what extent the project allowed citizens in rural areas of Tanzania to express themselves, reach key stakeholders, and to hold them accountable. In the last section, we will give concluding remarks and recommendations on using big data in development sociology.

## Big data in development research: A brief overview and its sociological potential

Big data provides new opportunities for international development research and evaluation and is receiving increasing attention (Abreu Lopes et al., 2018; Data2X, 2019; York and Bamberger, 2020). Big data are characterized by their remarkably large volume, variety, and velocity—big data is enormous, comes from different sources and in both structured and unstructured formats, and the data flows at a fast pace and is often generated continuously (Salganik, 2018, Chapter 2). Following York and Bamberger (2020, p. 10) three categories of big data can be differentiated: (1) “human-generated (centered) data” including social media data, internet searches, and text data; (2) “administrative (transactional) data” including migration reports, employment data, and combinations of different governmental and non-governmental data sources; (3) “geospatial data” including “satellites, drones, and remote sensing”. Such data is created and collected by humans, companies, and governments for purposes other than research and require repurposing (Salganik, 2018, Chapter 2). Our own example study presented below uses Twitter data, thus falling into the first category.

Big data can be relevant for development research and especially impact evaluation—assessing the difference a specific intervention makes in people's lives—in at least two ways. First, in addition to other sources of data gathered through surveys or focus groups for example, big data can be used to evaluate the effects of social interventions. Individual interviews (face-to-face or on the phone), which can be both semi-or fully structured, are currently the standard for evaluation in international development. However, they are prone to many forms of biases, including social desirability bias (Krumpal, 2013), and the frequent use of interviews can lead to respondent fatigue. Table 1 provides a comparison of big data and standard survey data regarding selected characteristics (see York and Bamberger, 2020 for a more comprehensive overview). It exemplifies that both big data and survey data have advantages and disadvantages. For example, while big data can easily cover a whole population for which data is available and can (repeatedly) be collected in a relatively short time, surveys are less prone to sample bias, and they are typically tailored to the specific research question at hand. Using big data comes with limitations placed by the platform accessed (and its application programming interface and terms of service), thus restricting a researcher's autonomy. While social desirability bias toward the researcher is often present in survey data, users are generally not tailoring their online activity toward a potential researcher. Still, it needs to be considered that individuals craft an online (public) brand or profile and/or that undesirable content is not even allowed on and thus moderated out by a platform. Overall, and given these different advantages and disadvantages, big data analysis is not expected to replace but to complement existing approaches in development research.

**Table 1:** Comparison of big data and survey data used in development research and evaluation.

**Category**	**Big data**	**Survey data**
Coverage	Often whole population for which data are available but can be limited due to platform constraints and data deletion. [+/–]	Sample size requirements, e.g., due to costs, limit coverage. [–]
Sample bias	Data can be selective (e.g., only social media users included). [–]	Selection bias can be controlled as part of sampling. [+]
Relevance for a specific research question/evaluation	Data often created for different purposes. [–]	Data created for specific research. [+]
Social desirability bias (toward the researcher)	Often not present. [+]	Can be present. [–]
Time for data collection	Short(er) time needed for data collection. [+]	Long(er) time needed for data collection. [–]
Longitudinal data	Easy to collect panel data. (+)	Difficult to collect panel data. [–]

Adapted from York and Bamberger (2020, p. 9).

Second, big data can be part of intervention programs: interventions conducted on or using social media, with their effectiveness subsequently evaluated. This way, (big) digital trace data can be more easily combined with causal analysis which is a crucial part of estimating the effectiveness of interventions. Our example study presented below falls in this second category.

To highlight the potential of big data for development research and sociology, in the following we briefly present five example studies employing different types of big data and focusing on sociologically relevant topics (see also Table 2). The first example is the study of Jean et al. (2016) in which they predict poverty in developing countries using satellite imagery and machine learning. Data can guide research and (political) decision-making to combat and prevent poverty. However, valid and specific data can sometimes be missing depending on the country's context: In particular, data may be incomplete and not capture all aspects of the multidimensionality of poverty. It is unrealistic that large-scale surveys can be used to compensate for this lack of data (e.g., due to high costs). Jean et al. (2016) use publicly available high-resolution daytime satellite imagery in combination with machine learning to obtain poverty and wealth estimates at the “village” level. To this end, they train a deep learning model based on a “noisy” but easily accessible measure of poverty: night-time lightning. As part of this process, their “model learns to identify some livelihood-relevant characteristics of the landscape” (Jean et al., 2016, p. 791). This approach was validated for five African countries (Nigeria, Malawi, Tanzania, Uganda, Rwanda).

**Table 2:** Five examples of Big Data used in sociologically relevant development research.

**Topic (and study)**	**Country context**	**Type of big data**	**Role of big data**
Poverty reduction (Jean et al., 2016)	Nigeria, Malawi, Tanzania, Uganda, Rwanda	Satellite imagery	Estimating poverty and wealth indicators
Digital gender gaps (Fatehkia et al., 2018)	Global (over 150 countries)	Facebook advertisement data	Predicting digital gender gaps
Educational inequalities (Khan, 2019)	Pakistan	Mobile phone data	Explanation of gender-based educational inequalities at the district level
Combating HIV in contexts of social stigma (Green et al., 2014)	Ghana	Facebook, WhatsApp and Badoo data	Part of an intervention to promote HIV testing and counseling
Social protest / revolutions (Koehler-Derrick, 2013)	Egypt	Google Search Data	Monitoring public opinion and attention where polls are biased

The second example is also concerned with existing data gaps; the study of Fatehkia et al. (2018) tracks the global digital gender gap. Such gender gaps are difficult to measure, especially in low-income countries. Fatehkia et al. (2018) use Facebook advertisement data on users by age and gender to predict digital gender gaps for over 150 countries. Facebook data is shown to be highly correlated with official data on digital gender gaps and their study is thus another important example showing how web data can expand coverage of development indicators (see also follow-up study Kashyap et al., 2020). In this line of research, several studies have discussed approaches to monitoring Sustainable Development Goals using big data (see for a review Allen et al., 2021).

The third example focuses on gender-based educational inequalities. Using mobile phone data from a large provider in Pakistan, Khan (2019) analyzes anonymized call detail record data comprising over one billion voice and text messages from approximately six million individual users. These data also include information on individuals' gender. Khan (2019) calculated district level averages for social network characteristics such as number of calls, network size, or friendship clusters. With a focus on gender differences, he then predicted gender-based educational inequalities in terms of primary school enrolment based on social network characteristics. He found that three network characteristics can explain almost 50 percent of the educational inequalities at the district level. These characteristics are “gender diversity of male calling networks”, “clustering of friend groups across all networks”, and “geographical reach across networks”. Data2X (2019) presents many more of this type of big data studies in development research.

The fourth example study refers to combating HIV among men who have sex with men in Ghana where same-sex sexual acts between men are criminalized and gay men face stigma. As part of this pilot intervention study by Green et al. (2014), three “communication liaison officers” were employed who reached out to the target group on social media platforms including Facebook, WhatsApp, and Badoo. The overall project also had a face-to-face component based on 110 peer educators. The project team managed to reach over 15,000 men of the target group *via* the social media approach and over 12,000 *via* the face-to-face approach. Both approaches seemed to increase HIV testing and counseling uptake with a 99 percent increase *via* social media and a 64 percent increase *via* the offline intervention. While this pilot study has several limitations, for example regarding recording actual contacts with HIV testing and counseling, it demonstrates the potential of social media to get in contact with hard-to-reach populations in contexts of strong social stigma. Also, in this case study, social media approaches are shown to be much more cost-effective than face-to-face approaches.

The fifth example employs Google Search Data to examine political developments in Egypt in 2011/2012 (Koehler-Derrick, 2013). The Google data indicates a sustained interest in revolutionary figures and actions which contrasts with reports by the Supreme Council of the Armed Forces. This can be seen as an example of how big data can help to uncover “true preferences” and public opinion when other data sources such as “official polls” provide biased results. Yet, Koehler-Derrick (2013) also points to the disadvantages of Google Search Data which ideally needs to be combined with other forms of data collection to validate findings. Furthermore, the use of such data is only meaningful in contexts with sufficient internet penetration.

Using computational tools, we argue that, for at least three reasons, sociology can make substantial contributions to better understand and explain development issues. First, as also indicated in Table 2, it is obvious that many development issues refer to core explananda of sociological analysis. Such issues include for example poverty reduction, tackling social and structural inequality, strengthening civil society, and promoting norm and cultural change. Big data related research on these issues can benefit from sociological insights on these substantive topics. Thus, it might be especially beneficial in inter- and transdisciplinary contexts, which is most often the case in development research. Second, sociologists make important contributions to computational social science research in general which can benefit research on development issues. Important areas include the study of social networks (e.g., group formation), collective action (e.g., social protest movements), sociology of knowledge (e.g., consensus in science), cultural sociology (e.g., processes of cultural change), economic sociology (e.g., the role of culture for economic transactions), and population studies (e.g., estimating migration patterns) (see Edelmann et al., 2020 for an overview). Here, analytical approaches in sociology might help to move from prediction to explanation (i.e., uncovering behavioral determinants and mechanisms) in development-related big data research. Third, the field of sociology of development is particularly strong in mapping and reflecting on the “development sector” including governmental and non-governmental actors, how their decision-making affects communities and individuals' lives (Viterna and Robertson, 2015), and the power dynamics at play. Regarding big data research, important topics include the link between knowledge and power, whose knowledge is valued, and how structural inequalities can be reproduced in and through (computational) research. Combined with a “digital sociology” perspective (e.g., Marres, 2017), sociology can help to shed more light on the interplay between the development sector, big data approaches/analysis, and community/individual material living conditions in so-called developing countries.

In the following, we present a case study on active citizenship and governance, an inherently sociological topic. As part of a larger development project, this case study also employs a social media intervention. It is therefore a more in-depth example of how (big) social media data can be integrated into a development intervention. With our case study, we want to highlight the potential use of social media data in action, highlight different avenues of analysis, and discuss its limitations.

## Case study: Active citizenship in Tanzania

In this section, we will introduce and present the findings of our study on active citizenship in Tanzania (Pretari et al., 2019). We will follow several different approaches to work with and analyze the (big) digital trace data collected to highlight the value-added and the limitations of this data in the context of development research.

This Oxfam-led project was implemented from February 2017 until March 2019 in four rural areas in Tanzania. The project aimed at improving community-driven governance and accountability through the use of digital technology. We will first introduce the project and its broader (theoretical) context in more detail before describing the methods and data used in this analysis. In this case study, the question we aim to answer is whether the intervention focusing on digital technology was effective at increasing greater online engagement. We analyze the online activity levels of those involved in this intervention and take advantage of the unique opportunity of assessing potential long-term effects. Particularly, we focus on the following key research questions: How have the animators and influencers involved in the project used Twitter over time (extent and content) and how was their content received by both key stakeholders and the general public? The following section will try to answer these questions. Across all analyses, the focus of our study lies on the sustainability of the intervention and changes across time. We want to make it explicit that we do not evaluate the overall project in this paper (see for this Pretari et al., 2019) but that we focus on the online Twitter component only.

### Project background

Oxfam in Tanzania launched the “Governance and Accountability through Digitalization” project in 2017. The project built on the traditional animation approach developed through a former project “Chukua Hatua” (“Take Action” in Swahili), namely community animators, village-level organizers, or facilitators who mobilize or *animate* communities around a common advocacy agenda. The former project was launched in 2010 and was implemented in five regions of Tanzania. By encouraging active citizenship, particularly for women, it aimed to achieve increased accountability and responsiveness of the government. According to the “Effectiveness Review” of the program—a series of impact evaluations conducted on a random sample of mature projects and commissioned by Oxfam—, it has made crucial contributions to its selected outcomes (it contributed toward making councilors more aware and responsive, toward citizens mobilization by animators, and toward gaining support for community forest ownership; see Smith and Kishekya, 2013). The “Governance and Accountability through Digitalization” project then enhanced the traditional animation approach by integrating digital tools. This project was developed and implemented in collaboration with three Tanzanian civil society organizations.

Oxfam itself has been working in Tanzania since the 1960s and has been aiming to ensure enhanced governance and transparency, women's empowerment, and to tackle rural poverty. This project is unique in integrating digital tools into this context of governance and accountability in Tanzania. Other development projects in Tanzania, which made use of digital technologies, have tackled issues regarding the job search costs in rural areas by introducing an SMS-based messaging application to connect agricultural workers and employers on wages and evaluating it using randomized trials (Jeong, 2021) or regarding violence against women by using (in an ongoing project) mass media campaigns to shift attitudes and behaviors (Green et al., 2018; the project is building on an earlier study in Uganda, see Green et al., 2020). Next to these studies making use of digital technology, other recent projects in Tanzania, for example, tested the impact of gender training interventions on intimate partner violence (Lees et al., 2021), of financial incentives for testing negatively for sexually transmitted infections to prevent HIV and other infections (De Walque et al., 2012), of handwashing and sanitation on child health (Briceño et al., 2017), of increased school resources and teacher incentives on student learning (Mbiti et al., 2019), or of financial incentives on female land ownership (Ali et al., 2016).

The “Governance and Accountability through Digitalization” project presented here took place within the setting of Tanzania's Cybercrime Act of 2015, which criminalized and penalized different cyber activities. This act has been criticized from the very beginning by civil society as a threat to freedom of expression and as a means to control online spaces. The project was implemented between February 2017 and March 2019, and these years have seen a shrinking of the civic spaces in East Africa and a change in the political climate in Tanzania. The Human Rights Watch World Report 2019 highlights that “since the election of President John Magufuli in December 2015, Tanzania has witnessed a marked decline in respect for free expression, association, and assembly”. In particular, the report highlights cases of criminalization of the sharing of information on WhatsApp, Facebook, or other online platforms by citizens and activists following the Cybercrime Act of 2015.

In this setting, the project built on traditional village-level animation approaches and enhanced them through the use of digital media. In our following analysis, we focus on Twitter. Internet and social media penetration in Tanzania has been increasing in recent years. Tanzania is undergoing a digital transformation with a growing number of people connected to communications and internet services (Okeleke, 2019). As of 2022, the (DataReportal., 2022) reports that 25 percent of Tanzania's 62 million inhabitants use the internet, while in 2017 when this project started, the number was 14 percent (DataReportal, 2017). 10 percent of the population is reported to use social media, and Twitter is used by 1 percent (DataReportal., 2022). No information on Twitter penetration is available for the past, but across all social media, 9 percent are reported to have used it in 2017 (DataReportal, 2017). In the case of the villages part of this project, 5 percent of women and 10 percent of men citizens reported owning a smartphone (Pretari et al., 2019, p. 51). We focus on Twitter as it is the platform which is most popular amongst the leaders, elites, and influential business leaders in Tanzania.

As outlined in the report of the former “Chukua Hatua” project (see Green, 2015), one of the main targets of the project has been to overcome the prevalent sense of powerlessness and futility in which citizens see no point in protesting or taking action as they expect no impact from it. The model/theory of change underlying the intervention holds that disempowered, marginalized people must feel a *power within*: people realizing they have rights and that those elected should serve them. This allows them then to build *power with*—the coming together of various forms of association around common issues—to achieve *power to*—asserting their rights, campaigning, and mobilizing. This exercise in active citizenship allows people to exercise *power over* key stakeholders. By promoting *power within, with*, and *to*, the project sought to enable people to raise their issues with those in authority and holding power, in whichever way they choose, including digital ones (see Pansardi and Bindi, 2021 for a critical account of the different concepts of power; in the present project it is conceptualized in relation to empowerment). Increased pressure from citizens for better delivery of public services is then expected to lead to local institutions being increasingly compelled to respond.

Using digital technologies to enhance animation approaches is also theoretically grounded in governance and social network approaches. The concept of *governance* includes more than the national government at the country level but includes the operation of formal power at national, regional, and local levels, as well as the way that informal powerholders influence those in power, and civil society engages with, and influences, formal powerholders (see Bevir, 2012 for a general overview). Good governance institutions are transparent and accountable to citizens, ensure that citizens' views and experiences are considered, and work to ensure that their needs are met (Smith, 2007; Rowlands, 2014). This project aimed to increase this self-awareness and power through online channels (see Criado et al., 2013 for a general discussion of the role of social media in governance). The internet brought new ways of socializing and instead of relying on closely-knit, location-based social ties, people moved into more fluid social environments (Wellman, 2001). This can enable new digital relationships with others that were previously unreachable: Digital platforms can thus be used to create new social ties.

Against this background, social media can become a way to raise local issues and join conversations, as well as mobilize other people and create online social networks. It builds people's capacity and skills so they can become active digital citizens. A further function of social media is that it allows obtaining (new) information (e.g., about one's own neighborhood). The project under consideration worked with animators and influencers to establish communication channels that facilitate the creation of and transition from power within to power with and power to. This can be further theoretically conceptualized as a form of *network governance* (Keast, 2022) and *social capital* creation (Lin, 2001). In this regard the animators and especially influencers function as brokers in a social (online) network (Kadushin, 2002) creating bridging social capital if authorities respond to citizens' demands. This is well in line with Putnam's (2000, p. 411) notion of bridging social capital in offline communities: “To build bridging social capital requires that we transcend our social and political and professional identities to connect with people unlike ourselves.” Further, as animators (more details below) are well embedded at the village level, they facilitate both bridging (weak ties) and bonding social capital (strong ties) at the village level. While network governance structures are more fragile than other forms of governance, they can be more effective, for example in the transmission of new information (Granovetter, 1973; Park et al., 2018), a key aspect of the “Governance and Accountability through Digitalization” project.

### Study design

Against the background of the Cybercrime Act and as a continuation of the former project, the “Governance and Accountability through Digitalization” project was launched in 2017. The project mobilized different actors, online and offline. The primary mechanism to achieve the project's aims relied on placing the power and information of the internet in the hands of roughly 200 community animators from four districts (in the regions of Arusha, Mtwara, Kigoma, and Geita) through the provision of smartphones and training workshops on the use of available associated technology such as search engines, WhatsApp, Facebook, Twitter, and other social media platforms, etc. These online mechanisms came in addition to offline interactions between animators and key stakeholders like government officials.

The selection process of animators was implemented by partners and supported by Oxfam. Villages were selected where at least a 2G connection was available with 3G being preferred, and the focus was on villages that had taken part in the previous “Chukua Hatua” project^2^. 62 villages were identified in addition to the Nduta refugee camp in the regions of Mtwara, Kigoma, Arusha, and Geita. In these villages, animators were selected using the following criteria (see also Pretari et al., 2019, p. 14):

- has taken part in animation activities (for Oxfam or other organizations),
- can read and write (this criterion may not have been met in very rare cases if the animator was very active and influential in the community),
- is not a political party leader, or involved in politics, nor a leader of the village/ward government,
- is a resident of the village/locality,
- is confident, can explain issues clearly, is concerned about issues and bringing about change in their locality.

While animators thus generally had prior experience in activism, only about 20 percent had used smartphones/social media platforms before. The project strategy relied on working with both women and men animators to consider gender dynamics and the fact that women citizens may feel more comfortable talking to other women, particularly on issues related to violence or discrimination, and ultimately ensure representation of women and men citizens' voices. A total of 50 animators per region were involved in the project. Partners settled on different strategies to determine the number of animators per village, and the number of villages involved. Ten villages are part of the project in Mtwara and five in the host communities in Kigoma, each with five animators. In Arusha, 25 villages are part of the project, with between one and four animators per village; in Geita, 21 villages are part of the project, with between one and six animators per village. It is important to note that these villages and regions have specific dynamics, are embedded in specific contexts, face specific governance issues, and feature a particular setup of animators. For example, in Kigoma, half of the animators were refugees fleeing Burundi who live in the Nduta camp, and the other half were members of host communities. In Arusha, animators lived in the Ngorongoro district, a district with long-lasting land disputes between the Maasai people, the government, and companies.

The project also sought to strengthen the link between local activism enhanced by digitalization through animators, and national influencing, through the mobilization of influential bloggers and social media users (later on referred to as *influencers*). These influencers were online users who had reasonable followership on social media platforms (amount of people who followed them; followed by leaders, high profile individuals, etc.) and whose social media posts were more likely to attract engagement from diverse audiences. Substantially, they are users who were posting mostly about issues/topics that are core to the human rights agenda and are using social media platforms for social good. Influencers thus had prior experience with social media use, but not all of them had prior experience in activism.

Animators and influencers were not paid to participate, but Oxfam provided the animators with mobile handsets and a monthly airtime allowance of 30,000 Tanzanian shillings (equivalent to 12 US dollars). They were also provided with solar chargers to charge their phones since most of them came from rural areas with limited or no electricity. In addition, Oxfam ensured there was ongoing technical support from local partners should animators need support using their digital devices. The most important incentive was the expectation and experience of receiving immediate responses and solutions from key policy makers and duty bearers on issues they had raised.

Participants used different social media platforms to engage with the community and duty bearers and there were further mechanisms employed to supplement the use of online platforms and offline activities. The different digital platforms were used differently. Twitter proved to be the most popular amongst the leaders, elites, and influential business leaders in Tanzania, making it useful to reach these key stakeholders. On the other hand, WhatsApp was effective for organizational tasks: WhatsApp groups were used by animators to coordinate, share information, chat on issues, and agree on topics and strategies before going public. WhatsApp also proved to be effective to reach duty bearers at the regional and district level in Arusha.

Radio programs facilitated debate between citizens and duty bearers, raising awareness on various issues related to human rights and social services. Weekly Twitter debates and regular YouTube live streaming sessions were held. Participants were required to take part in these weekly debates and use the hashtag #*ChukuaHatua* to highlight various community challenges and demand responses and actions from policy makers and duty bearers. These live streaming sessions provided an alternative to mainstream media, as well as a link for the community animators from rural and urban areas to share their experiences. The social media influencers played a key role in capturing the attention of the public during these events.

Throughout the project timeline, animators were provided with introductory and refresher trainings (and certificates of attendance) on animation and on how to effectively use digital tools to raise issues that are important to their communities. In the training workshop on digital tools, animators received smartphones and were shown how to use them and their technical features like the camera to take photos and videos, as well as how to make use of existing social media platforms like Facebook, WhatsApp, and Twitter. Participants were also required to establish a social media strategy—they selected issues that were relevant to their communities and developed a work plan and timeline to address those issues. They created an issue-based calendar stating in which month they planned to focus on which topic. This was also the case for influencers who tweeted about the selected topic during the weekly debates. Participants were further trained on the relevant laws governing digital platforms. They were taught to understand key contents of the Cybercrime Act and were encouraged to post within the guidelines of the act. The influencers received this training from officers of the Tanzania Communications Regulatory Authority who are the key implementers/overseers of the act. Additionally, a sensitization workshop took place with civil society partner organizations and leaders/officials (at village, ward, district, and regional levels).

### Methods and data

In the following section, we will provide details on the Twitter data collected and the statistical approaches used in our case study. The analyses presented here are building on the previous Oxfam impact evaluation (see Pretari et al., 2019) where we retrieved and analyzed over 130,000 tweets at the end of the official project timeline (March 2019). We extend this now and particularly focus on the long-term sustainability of this developmental intervention. Having this possibility is a unique advantage of online data in comparison to other data sources in developmental research which we want to highlight and explore in the following analyses.

#### Data collected

For our analysis, we collected Twitter data from all ~200 animators and influencers involved in the project to analyze both, the animators' and influencers' behavior during the implementation of the project, and the potential long-term effects.

In the past, research efforts on Twitter were severely limited due to restrictions imposed by the application programming interface (API). In 2021, a new academic research track was launched by Twitter, allowing an expansion and improvement of data collection. This allowed us to now collect the complete Twitter timeline of all animators and influencers involved in the project since the creation of their accounts. We thus follow an elite-centered approach when collecting data, focusing on specific user accounts. Data was collected using the R-package *academicTwitteR* (Barrie and Ho, 2021).

It was attempted to collect the complete Twitter activity of all of the 194 animators and influencers (see Table 3 for descriptions of the users and tweets collected). However, some Twitter handles referred to profiles that did not exist, so user data of only 181 profiles was retrieved. While this only affected a small number of Twitter accounts in most regions, almost one fifth of accounts are missing for Kigoma, which might influence the data if these are systematic losses (for example, it might be that primarily non-active Twitter users have misremembered their Twitter handle). Past tweets were retrieved from a total of 168 Twitter users. Most tweets in the complete dataset come from the 24 influencers (98 percent). The 13 users for which no tweet data could be obtained either had a private profile, which could not be accessed, or have never tweeted. The oldest tweets for the animators and influencers date back to May and June 2009, respectively. The most recent tweets are from October 2021 which was set as the limit during data collection.

**Table 3:** Description of user and tweet data.

		**Mtwara**	**Geita**	**Arusha**	**Kigoma**	**Influencer**	**Total**
User-level information	Users on list	50	48	42	26	28	194
	Users with retrievable profile information	49	45	41	22	24	181
	Users with retrievable tweets	45	42	39	18	24	168
	Retrieved users' average followers count	160.6	95.4	54.0	228.7	49,358	6,652
	Retrieved users' average following count	150.5	166.1	72.8	520.5	3,245.5	592.1
	Users who received replies from officials	7	1	0	5	14	27
Tweet-level information	Tweets posted (excluding retweets)	10,626 (2,761)	5,378 (3,606)	1,497 (1,171)	10,649 (4,539)	1,575,567 (925,568)	1,603,717 (937,645)
	Hashtag used	23.4%	21.3%	30.4%	36.3%	18.2%	18.3%
	User mentioned	90.3%	78.9%	91.8%	70.8%	66.4%	66.7%
	Mentioned *chukua hatua*	2.9%	4.2%	6.3%	4.0%	0.13%	0.19%
	Average engagement received (excluding retweets)	1.9 (1.5)	1.2 (0.79)	1.2 (0.77)	1.8 (1.3)	1.4 (0.71)	1.4 (0.72)
	Replies received	10	1	0	11	579	601

We also collected Twitter data on relevant officials in Tanzania. These relevant officials include national level leaders [such as (prime) ministers, the vice president, and the president], local level leaders (on the level of the village, district, and region, as well as councilors and members of parliament), institutions relevant to the project (such as the public electricity company, the communications regulatory authority, surface and marine transport regulatory authority, the ports authority, or the national bank), non-governmental organizations and Oxfam partners. The list of officials included 56 user accounts and was created by a subject matter expert (the complete list can be found in the Appendix). We were able to retrieve profile information of 49 and tweets of 48 of those accounts.

#### Methods

We will employ several different analytical strategies to shed light on the (long-term) effectiveness of the intervention. To answer our research questions, we first describe the extent of activity across time focusing on the animators' and influencers' Twitter usage from the start of the intervention. We then describe the content of all their tweets and identify the topics covered by counting the most frequent words. The majority of tweets collected are written in Swahili which makes it difficult to use more advanced out-of-the-box solutions to address natural language processing tasks, as these solutions are most often based on English texts.

The focus of our descriptive analyses lies on changes throughout time. During the project, Oxfam conducted several workshops to train their participants on the use of digital media. Thus, after the description, we assess whether these workshops were impactful. We focus on a refresher workshop on digital tools which took place on 4 weekends in July and August 2018, each weekend taking place in a different region. This allows us to make use of a difference-in-differences-design (DiD) (Angrist and Pischke, 2008). DiD is one of the most common approaches for identifying and estimating the causal effect of experiencing a treatment on some outcome.

In the canonical DiD, two groups, a treatment group (T) and a control group (C), are compared across two points in time, before treatment (pre) and after treatment (post). In this setting, the simple DiD estimator is the difference between the differences in the treatment group and the differences in the control group:


(1) $$\begin{array}{l} {\hat{\delta }}_{DiD}=E\left(\Delta y_{T}\right)-\;E\left(\Delta y_{C}\right)\\ =\;\left(E\left(y_{T}^{post}\right)-\;E\left(y_{T}^{pre}\right)\right)-\;\left(E\left(y_{C}^{post}\right)-\;E\left(y_{C}^{pre}\right)\right) \end{array}$$

In this setup, the untreated group never participates in the treatment and the treated group receives the treatment in the second period. In cases of more than two time periods and different treatment times for different units, the leading approach to estimate the effect of the treatment is to use a two-way fixed effects linear regression. However, a number of recent methodological papers have raised concerns about using the two-way fixed effects model with multiple time periods. Particularly, the model is shown not to be robust to treatment effect heterogeneity (De Chaisemartin and d'Haultfoeuille, 2020; Goodman-Bacon, 2021; Sun and Abraham, 2021). To tackle this issue, Callaway and Sant'Anna (2021) have proposed the use of a flexible DiD. They generalize the 2 x 2 DiD in a multi-group and multi-timing setting by computing group-time average treatment effects. With this approach, individual treatment effects for each combination of treatment-timing-group and control group (either never-treated or not-yet-treated) are estimated. These different treatment effects are aggregated in a second step to a group- or time-averaged treatment effect. The model assumes staggered treatment adoption, parallel trends, and no treatment anticipation. Applied to our context, this means that the animators based in Mtwara form the first treatment group and they are then compared to those in the other regions (as they have not taken part in the workshop yet, in other words they are “not-yet-treated”). Each region is thus then compared to the other groups. The dependent variable in our first dynamic DiD is the number of tweets sent; a measure of activity and the DiD thus measures whether tweeting activity increased after the refresher workshop.

In the next step, we focus on engagement received instead of activity, providing insight into how the level of engagement changed over time. First, we analyze the general popularity of the animators' and influencers' self-written tweets. Retweets are excluded as they do not represent the animator's and influencer's content as clearly (they might be retweeting more popular tweets). Again, we assess how this has varied over time and whether it was causally affected by the refresher workshop employing a dynamic DiD. Engagement is then defined as the sum of the retweet and the like count of tweets. Next, we focus on a second type of engagement: that between the project participants and key stakeholders. As this is a very different context, we focus on replies. A reply on Twitter is a time-stamped response to another tweet. It is a way to join a conversation. While follower-followee relationships can be a measure of popularity (using the follower relationship see e.g., Verweij, 2012; Hofer and Aubert, 2013), and retweets can act as a signal of endorsement (using retweets see e.g., Conover et al., 2011a,b), replies are a measure of active interaction (Sousa et al., 2010; using replies see e.g., Bliss et al., 2012; Gaisbauer et al., 2021). A follower-followee approach is less meaningful in our setup as official accounts (such as accounts of institutions) do not tend to follow (many) others. There were 702 instances in which officials replied to animators or influencers. Most of these replies (676) have been toward one of 14 influencers. Only a few animators per region have been in conversation with officials (see Table 3; please note that while we find 702 instances in which officials replied, we only find 601 undeleted, accessible, and unique tweets which have received a reply)^3^.

We use this information on key stakeholders to create a social network between officials and animators. We create an undirected, two-mode network with ties from reply-sending key stakeholders to reply-receiving animators/influencers. Again, we are making use of the longitudinal nature and compare social networks at different points in time (before the workshop, after the workshop, after the end of the intervention. We plot the networks and describe basic characteristics, making use of the R-package *igraph* (Csardi and Nepusz, 2006).

As a last part of our quantitative analysis, we go beyond description and ask which features of tweets are important in generating, on one hand, engagement with the general Twitter public, and, on the other hand, a reply from key stakeholders. We employ logit models to investigate these questions. Our data source to explain engagement are all tweets of animators and influencers which are not retweets (*n* = 937,645) while we work with the complete set of tweets for the latter analysis (*n* = 1,603,717). The level of engagement tweets receive varies greatly (mean 8.93, SD 108.94, minimum 0, maximum 70,452) and the majority of tweets receive no engagement at all (58.9 percent of tweets). As it is not our goal to focus on explaining what goes viral (see for such analyses in other contexts for example Zadeh and Sharda, 2022 or Pressgrove et al., 2018) we simplify our analysis by asking which tweets receive any engagement at all and thus create a binary measure. Receiving replies by officials is a rare occurrence; *n* = 601 (retrievable) tweets have received at least one reply (see Table 3).

In our models, we ask whether tweets referencing the project are particularly successful; to do this, we focus on the key term *chukua hatua—*the term is used as a hashtag to unite those active on Twitter and its usage has also been promoted through the Oxfam-led workshops. In line with our other analyses, we are further differentiating three project phases to assess to what extent engagement has varied throughout time and in the long term. We additionally include an interaction effect between the project phase and the project term *chukua hatua*. This setup will allow us to assess whether such strongly topic-related related tweets receive engagement from the public and key stakeholders and whether a possible effect of this project-relatedness has varied throughout time. We also test to what extent key stakeholders were more likely to reply to posts that were important to the public (by including logged engagement as an independent variable). To check the robustness of the keyword effect, we control for user- (whether they were an animator or an influencer, their geographic region, their popularity and activity measured as logged numbers of followers, followees and previous tweets) and tweet-level features (whether specific technical features were used, i.e., hashtags and mentions). Given that one participant has generally made multiple tweets, we employ cluster robust standard errors. We estimate four different models for both dependent variables. We run a set with and without control variables. In the first one, we include the total dataset; in the second model, we focus on the tweets posted after the beginning of the project (reducing the dataset to *n* = 430,866 for engagement, and *n* = 941,764 for replying behavior with *n* = 242 replies).

### Results

We present the results of our analysis in the following subsections. We first focus on the tweets themselves over time—their quantity and their content. In a second step, we focus on engagement with tweets.

#### Activity on Twitter over time

**Figure 1:**
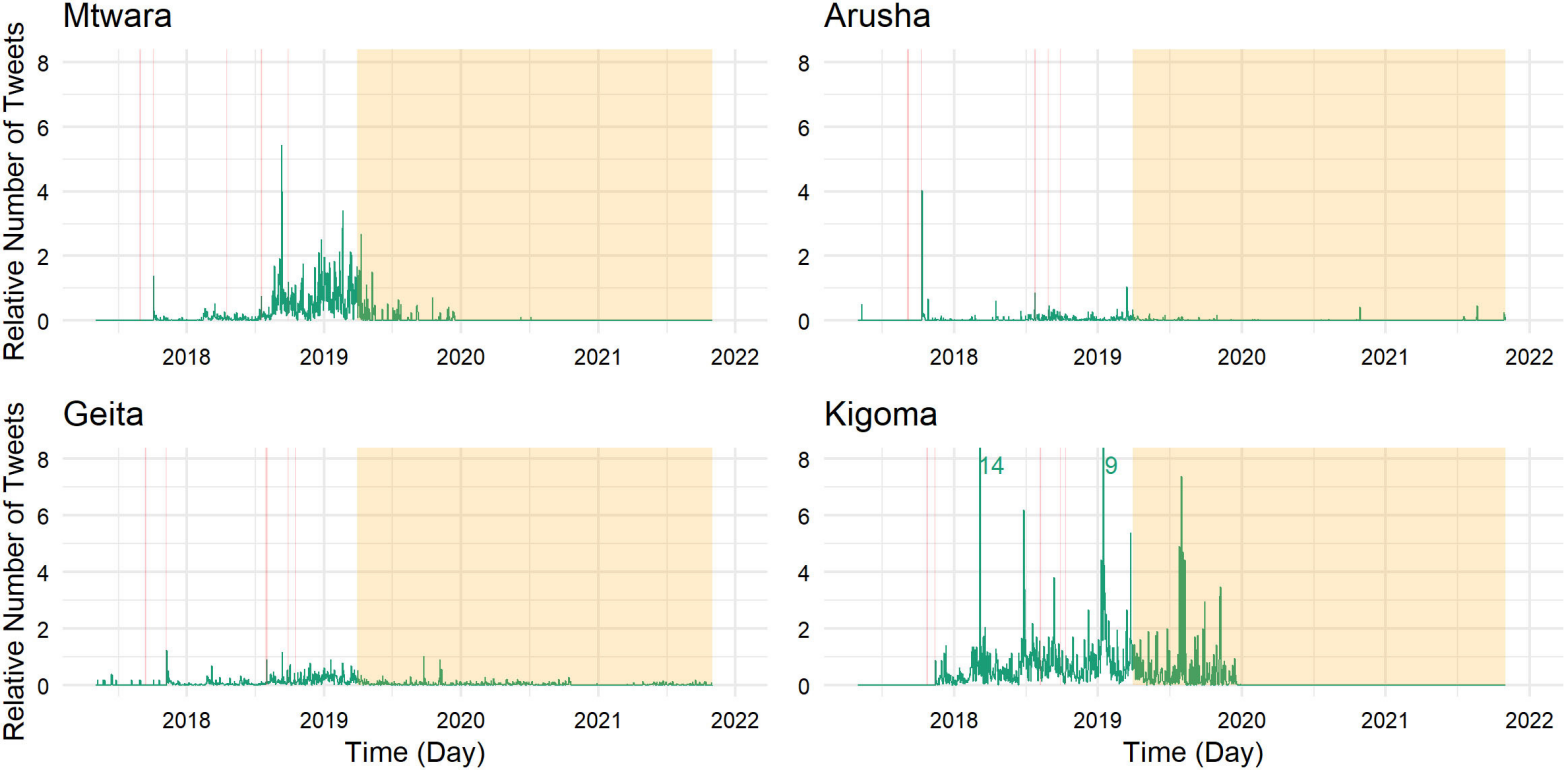
Twitter activity of animators across time per region.

**Figure 2:**
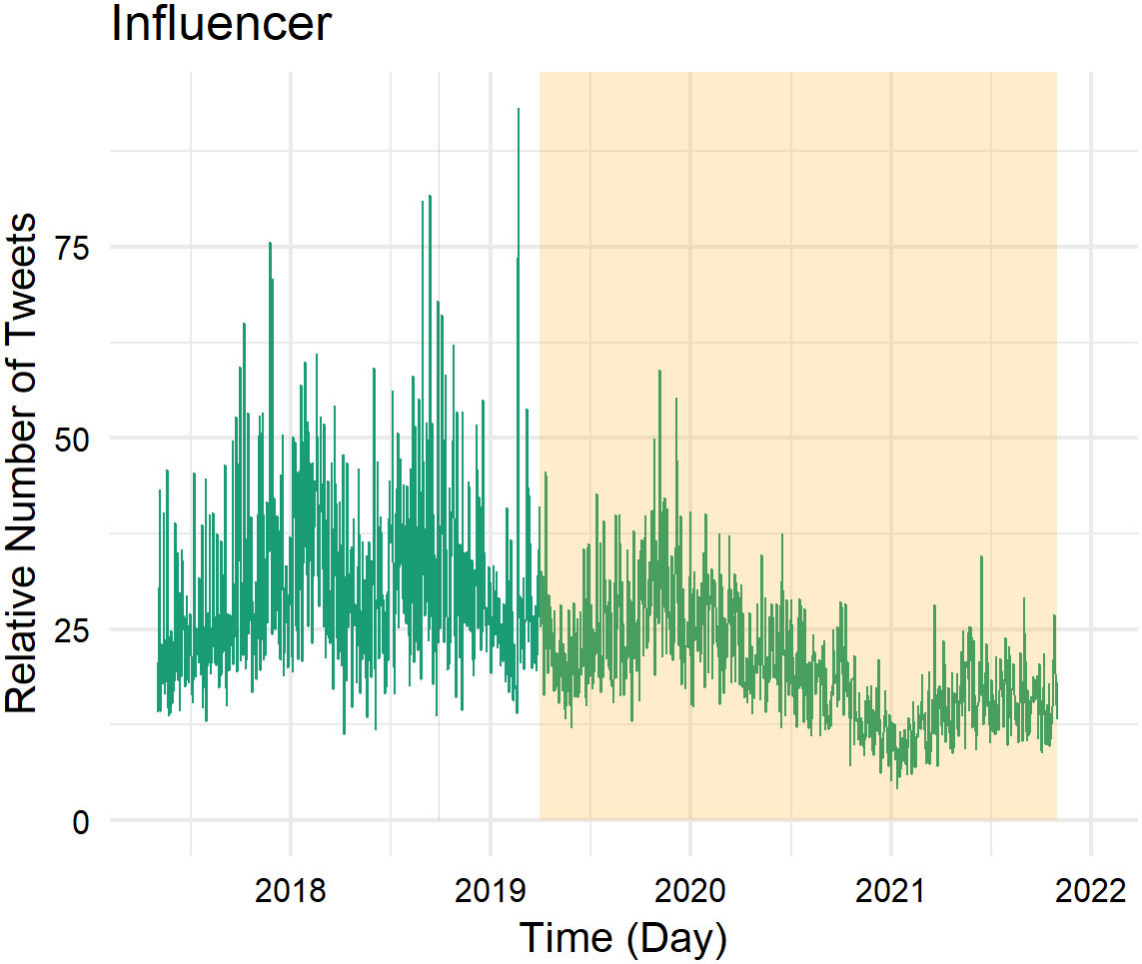
Twitter activity of influencers across time.

Figure 1 shows the relative frequency of tweets of the animators (number of tweets per day divided by the number of registered Twitter users) in all of the four regions since May 2017; Figure 2 focuses on influencers only. Areas shaded in red refer to days of training, workshops, or summits organized within the setting of the project. All animators took part in a training on animation, a training on digital tools, and a refresher workshop. The area shaded in orange highlights the time without airtime support (after the end of the project on 31 March 2019).

In the case of the animators, tweeting behavior is clearly spurred by an Oxfam workshop. During the workshop on digital tools, smartphones were handed out to the animators, and they were instructed on how to use them and social media, which had a clear effect on Twitter activity: Before this workshop in October/November 2017, we observe next to no tweets.

During the time frame of the project, we observe levels of tweeting activity that are rather stable, but comparably low in Geita and Arusha. More activity is observed in Kigoma and Mtwara. These regions also show large variations in the frequency of tweets and some remarkable peaks in activity. Airtime support ceased at the end of March 2019, but tweeting continued. Both in Kigoma and Mtwara, animators were still active up until the end of 2019 (more so in Kigoma). In Arusha, only a few scattered tweets are observable since the end of the project. Geita is an exception to the other regions where continuing tweeting activity is observable, however to a lesser extent than before.

Focusing on the influencers, the pattern of activity looks very different (see Figure 2). The influencers are generally much more active, sending on some days almost 100 tweets (per influencer). They have joined the Oxfam project as active tweeters and have thus already been registered and tweeted before the project started. Their tweeting activity is thus expected to cover much more than just the project's time length. We observe a reduction in their tweeting activity in the recent past, especially starting the second half of 2020. Even though they reduced their activity, there is still no day in which they do not post any tweets. However, from the descriptive image, we do not observe any project-related changes.

**Figure 3:**
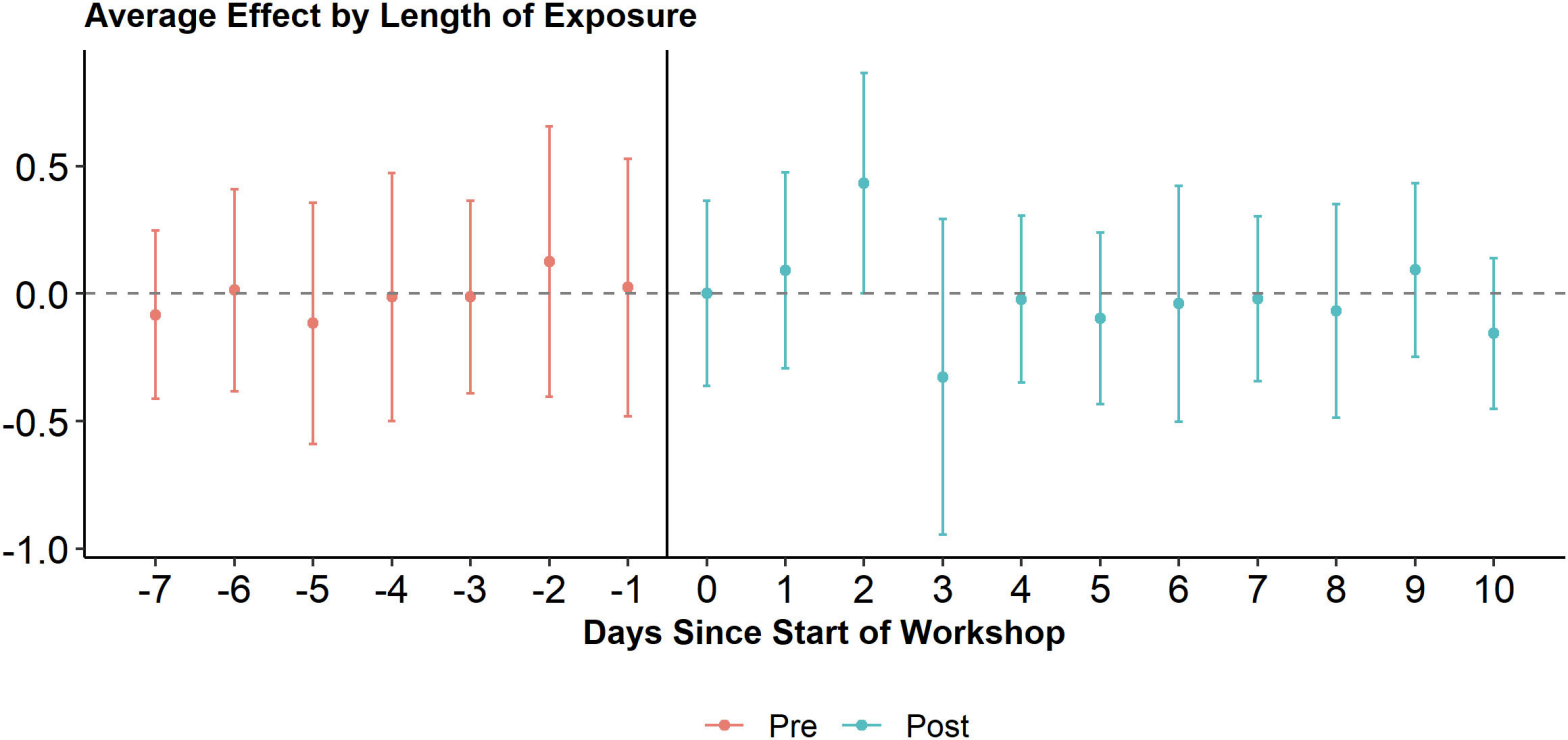
Average effect of Oxfam workshop on tweeting activity of animators. Including 95 percent confidence interval.

While the introductory workshop on digital tools sparked the animators' online behavior, what was the effect of the refresher workshop they received around 10 months later? We employ a dynamic DiD to assess its effect (see Figure 3). The Oxfam-led workshop took place over the course of a weekend (three days) and the first day of the workshop is considered the first day *post* treatment (time 0). We find no significant effects of the workshop on tweeting activity. On the third day after the treatment (i.e., the last day of the workshop), tweeting activity tends to increase on average, while on the fourth day after the treatment (i.e., the first day after the workshop) tweeting activity tends to decrease, but these changes are not significant on a five percent level. Overall, tweeting levels seem rather unaffected by the workshop. However, it is important to note that we cannot sufficiently take into account regional differences. The four regions in this project did exhibit very different dynamics, making comparison difficult.

Going beyond the quantity of tweets made by animators and influencers, we also analyzed their content. The 10 most frequent words per project phase (differentiating four phases: before the project started, before the refresher workshop, after the refresher workshop, after the end of the official project) and per participant type are shown in Table 4. As shown before, animators have generally not been active on Twitter before the project started and no frequent words are retrieved. Since then, both animators and influencers have used Twitter. Both groups mostly tweet in Swahili and to a lesser extent in English (influencers use English more frequently than animators). During the project, animators often tweet about and explicitly mention their region or district (Kibondo, Mtwara, Kigoma) and often talk about the situation in their *village*. Influencers, on the other hand, more broadly mention the country context of *Tanzania*. In the first phase, animators discuss issues around the *government, work, citizens*, and *school* most often. In the second phase, both animators and influencers start to more frequently link and refer to the project by using the term (and hashtag) *chukua hatua*. Both groups also more frequently discuss issues around *water*. After the project, *chukua hatua* is not the most frequent word mentioned, but still belongs to the 10 most frequent words used in the group of animators; influencers, however, do not refer to the project that often anymore. For animators, references to the regions also seem to have become less, while the terms *vegetables* and *development* have increased in frequency. Over the timeline covered, influencers discuss a variety of topics: Across all time frames, they also often raise issues around *work*, the *government, society*, and parts thereof like *children* or *young people*. *Elimikawikiendi*, a term often occurring as a hashtag, was used during the popular, weekly Twitter session (organized by a company^4^); the hashtag united various Twitter users. Participating influencers and animators used this to share issues of concern from their localities.

**Table 4:** Most frequent words per group and project phase.

**Group**	**Project phase**			
	**Before project**	**First phase**	**Second phase**	**After project**
**Animators**	No words occurred more than 5 times.	Kibondo Kijiji (the village) Serikali (government) Kazi (work) Kigoma Wananchi (citizens) Mtwara Shule (school) Tunaomba (we pray) Waraghabishi (animators/community activists)	Chukuahatua (take action) Serikali (government) Kijiji (village) Maji (water) Mtwara Wananchi (citizens) Kibondo Waraghabishi (animators/community activists) Jamii (society) Kazi (work)	Mbogwe (vegetables) Kazi (work) Serikali (government) Jamii (society) Wilaya (district) Maendeleo (development) Wananchi (citizens) Chukuahatua (take action) Maji (water) Kijiji (village)
**Influencers**	Tanzania Elimikawikiendi Rais (president) Kazi (work) Mkuu (principal) People Jmaa (relatives) Maana (meaning) Mama (mother) Time	Elimikawikiendi Tanzania Twittergulio (Twitter) Kazi (work) Serikali (government) Watoto (children) Rais (president) Mtoto (child) Mkuu (principal) Nchi (country)	Tanzania Chukuahatua (take action) Maji (water) Kazi (work) Elimikawikiendi Serikali (government) Vijana (young people) Watoto (children) Mwaka (year) Jamii (society)	Tanzania Kazi (work) Vijana (young people) Elimikawikiendi Mzee (old man) Aatoto (children) Maana (meaning) People Mwaka (year) Mtoto (child)

#### Engagement with the public and key stakeholders on Twitter over time

In a digitalized world, using social media has become a way to raise local issues to the public and it allows joining conversations with duty bearers at the national, regional, or district level. In this section, we will describe the changes in the public's and the key stakeholders' engagement with the tweets over time and address the question of which kind of tweets are most likely to receive engagement and replies.

**Figure 4:**
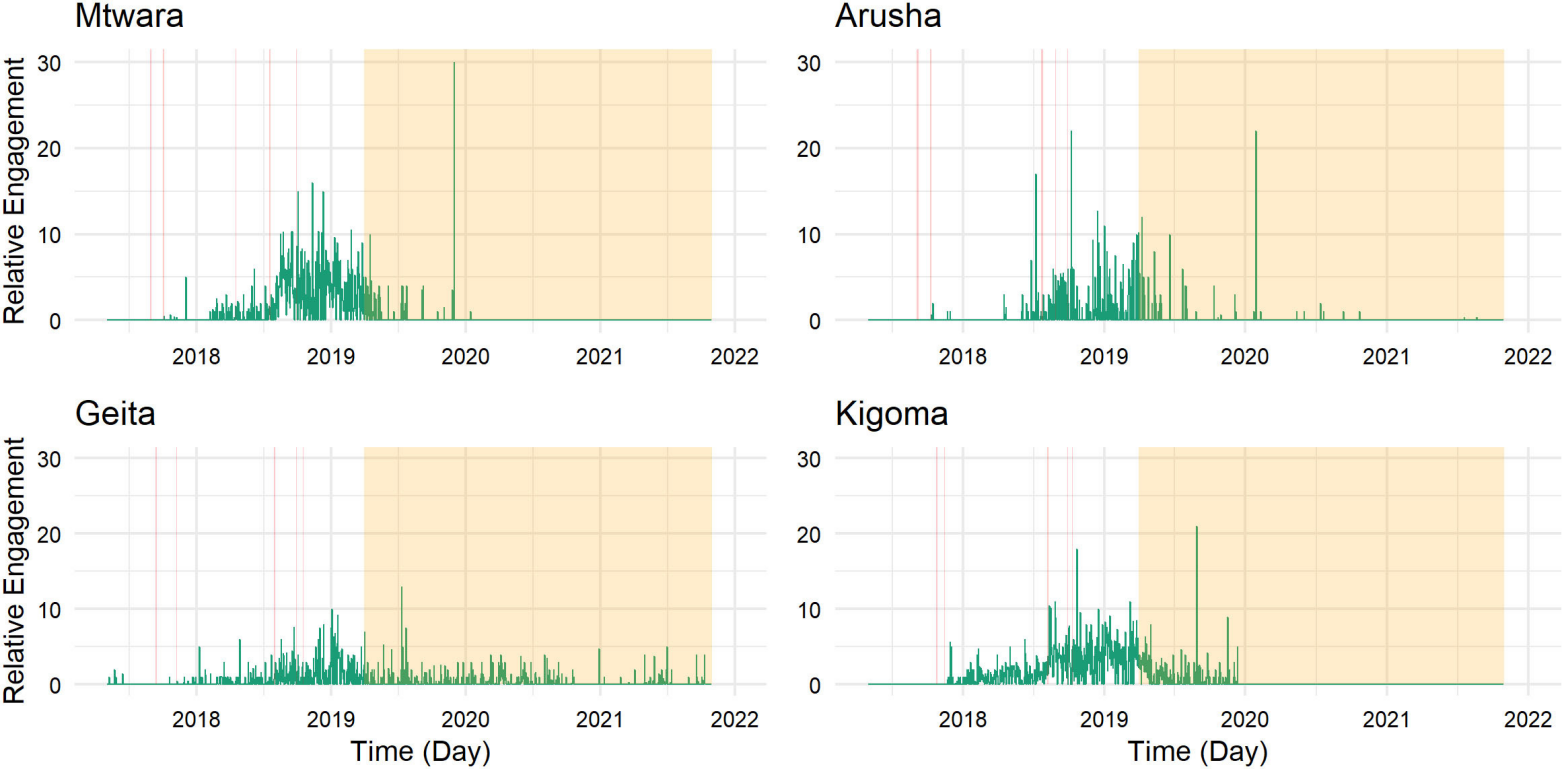
Twitter engagement received by animators across time per region.

**Figure 5:**
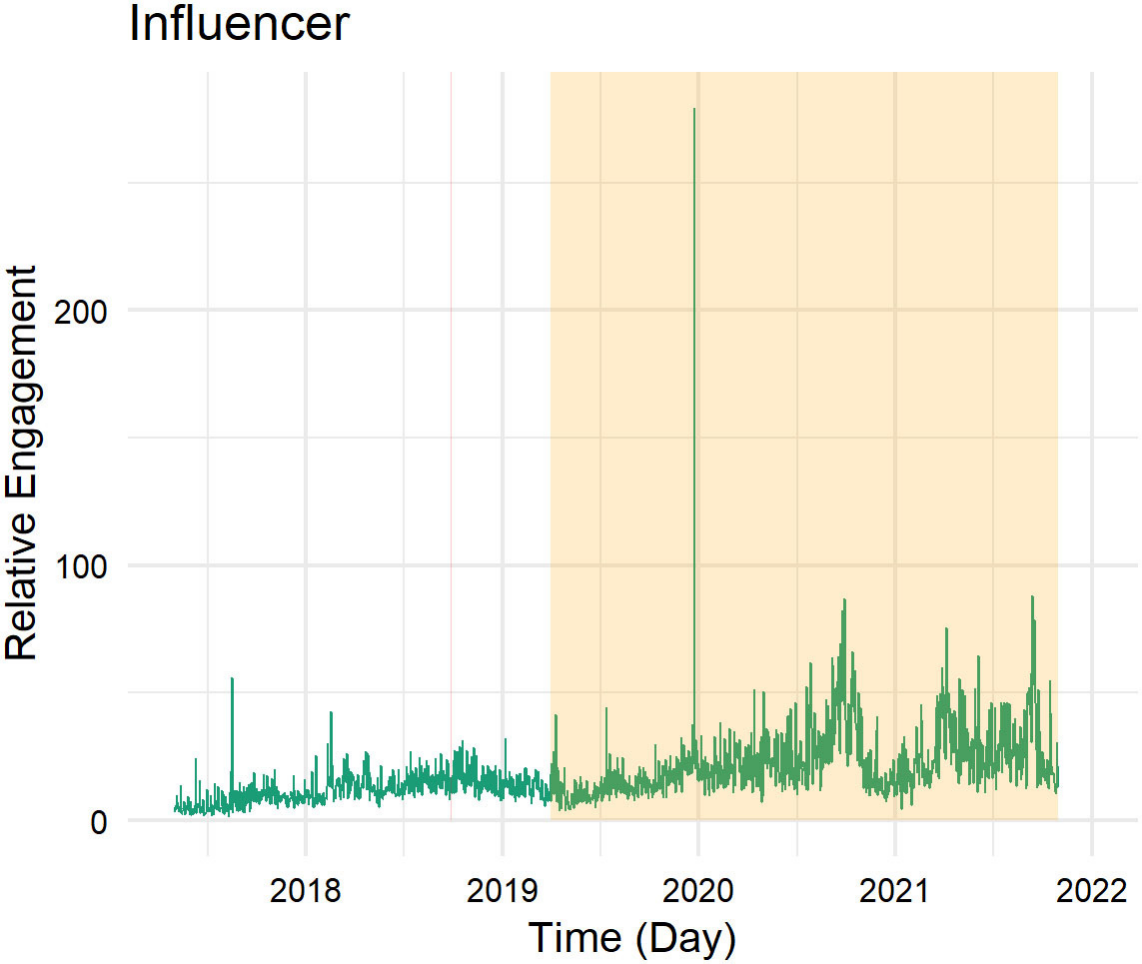
Twitter engagement received by influencers across time.

Using the same labeling as in Figures 1 and 2, Figures 4 and 5 display the relative levels of engagement for the tweets (excluding retweets) of the animators (count of retweets and likes per day divided by the number of tweets posted on that day) in the four regions (Figure 4) and focusing on the influencers (in Figure 5) since May 2017. This provides insight into how the popularity of the content put out by the animators and influencers changed over time and extends the previous section which focused on the volume of posts.

In the case of the animators, the level of engagement per tweet was highest in all regions in the later phase of the project (after the refresher workshop). This might suggest that their tweets might have become more effective in generating the public's interest after the workshop. While fewer tweets were made when the project ended, those few tweets still received the highest levels of engagement. The animators in Geita are the only ones still regularly tweeting, and their tweets still receive average levels of engagement from the public.

In terms of posts made by influencers, we observe a relatively stable level of engagement during the project's timeline, and an increase with some notable peaks since (see Figure 5). Influencers' tweets have received more engagement since the project ended. It might be the case that the topics they have tweeted about in the more recent past are more engaging and more popular, but it might also be that, as the quantity of tweets has decreased, only the more successful influencers are still active on the platform.

**Figure 6:**
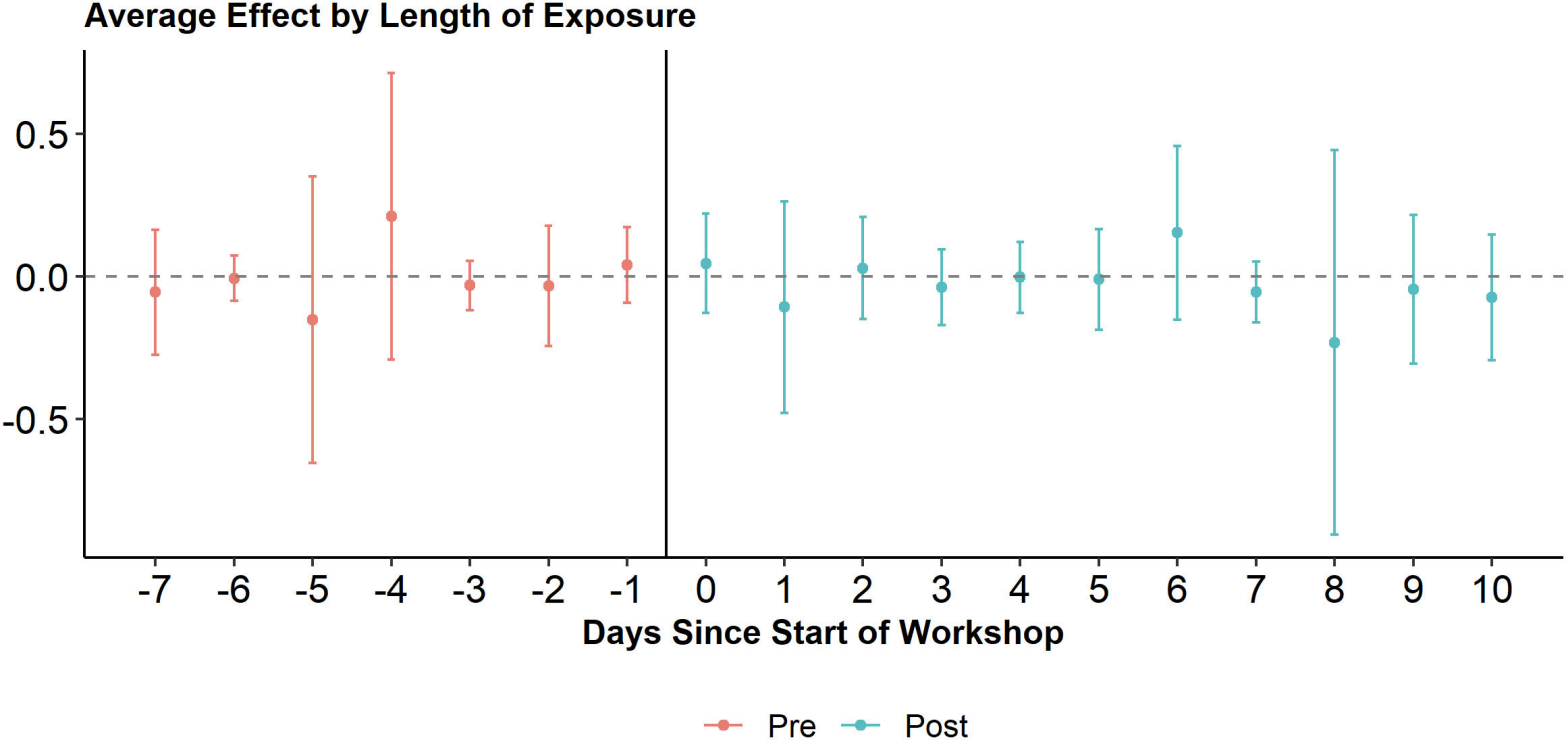
Average effect of Oxfam workshop on engagement received by animators. Including 95 percent confidence interval.

Figure 4 suggests that tweets by animators generate increasing levels of engagement in the long term after the refresher workshop. To test a potential causal effect of the workshop more explicitly and directly, we again make use of a dynamic DiD (see Figure 6). We use the same approach as when analyzing the workshop's effect on tweeting activity. We find no significant effects of the workshop on the average amount of engagement tweets receive: They seem unaffected by the workshop.

While the previous analyses have focused on the endorsement of tweets from the public *via* liking and retweeting, we will now focus on users' ability to connect with key stakeholders such as government officials, public service providers, non-governmental organizations (NGOs), and civil society organizations (CSOs) on Twitter. We are again making use of the longitudinal nature and compare social networks built through the reply function at different points in time: We take (A) all tweets from May 2017 up to June 2018 (refresher workshop), (B) all tweets from July 2018 up to March 2019 (end of the intervention), and lastly (C) all tweets since April 2019.

**Figure 7:**
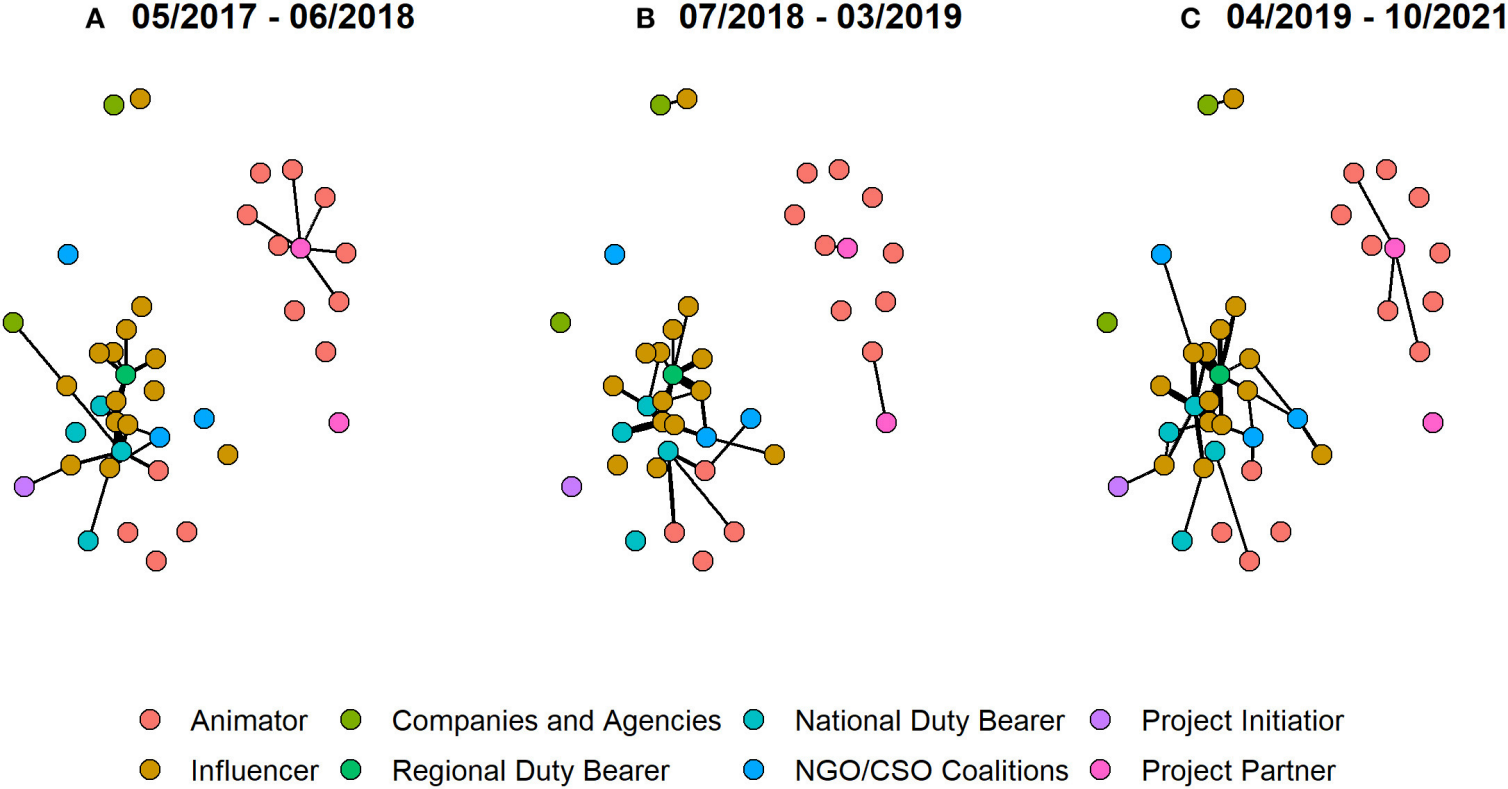
Reply network between officials and animators/influencers across three different time frames. Part **(A)** refers to tweets which were posted during the first phase of the project (May 2017 to June 2018), part **(B)** refers to tweets of the second phase of the project (July 2018 to March 2019), and part **(C)** refers to tweets posted after the project ended (April 2019 to October 2021).

The three networks are shown in Figure 7. Thicker edges reflect multiple replies. In the time frame from May 2017, 40 different nodes are part of the reply network (across the complete time span, there are 43 nodes). In network (A), these nodes share 141 edges; in network (B), they share 53 edges and in network (C), they share 101 edges. The number of edges shared between two nodes can vary as multiple edges are allowed: In (A), one influencer has received 42 replies from a national leader. The maximum of replies exchanged between the same two actors in a timeframe are 9 and 11 in time frames (B) and (C), respectively.

In the earliest timeframe, the network is split up into two components (containing 18 and 7 nodes) while the other 15 are isolates. After the refresher workshop, in timeframe (B), we observe 2 larger components containing 14 nodes and 5 nodes, respectively, 3 dyads, and again 15 isolates. Officials have still replied since the end of the project as seen from network (C) which is made up of 2 larger components (containing 22 and 4 nodes respectively), 2 dyads, and 10 isolates.

Across all time frames, the largest components refer to 13 different influencers and 4 animators which were in exchange with several different officials. Particularly, many replies were exchanged between three different national politicians and the influencers and animators surrounding them. They are pictured in the bottom left of the plotted networks. One regional duty bearer in particular has been replying to many tweets posted by influencers. While we cannot argue that this is caused by the refresher workshop—given that the number of replies is generally very small, and we look at long time frames with many unobserved characteristics—we do observe an increasing number of actors being involved in this discussion network across time and that these discussions have not stopped when the project did. Beyond the cluster in the bottom left, the nodes in the top right capture the fact that Oxfam and its partners have been in conversation with a number of animators. The separated dyad in the upper left corner reflects conversations between an influencer and an NGO or CSO coalition.

Since May 2017, 27 animators and influencers have received replies from official accounts; all others have not. Which tweets are most likely to receive replies? And which tweets receive the most engagement from the public? We have tried to explain whether tweets receive any engagement and whether they receive replies by officials using logit models as described in section Methods.

The results are shown in Table 5. Models 1.1.1 and 1.1.2, which only include tweets posted since the start of the project, suggest that tweets made in the later phases of the project (second phase after the refresher workshop and after the project as a whole) are more likely to receive any engagement (at least on the 10 percent significance level). Using the term *chukua hatua* makes tweets more likely to attract engagement, even more so in the second phase of the project (model 1.1.1). However, this is only observable when not controlling for other user- and tweet-level features (model 1.1.2). There, we only observe that tweets mentioning *chukua hatua* and sent after the project are less likely to receive engagement. Across all observations (models 1.2.1 and 1.2.2), we see that tweets made before the project started are much less likely to receive any likes or retweets than those sent in the first phase, and that, again, those made in the second phase or after the project are, on average, more likely to receive engagement from the public. Working with the complete set of tweets now, we observe a positive project word effect even when controlling for other features. Over time, making use of the project word seems especially positive in the second phase of the project, but can have a negative effect after the project (model 1.2.2 only). These patterns suggest that since the project started and also since the first phase of the project, engagement with tweets from the general public has increased. Making an explicit reference to the project is generally positive (positive main effect of mentioning *chukua hatua*) during the project timeline, but not since (negative interaction effect of mentioning *chukua hatua* and after project). Our control variables suggest differences between animators and influencers, regional differences, and strong effects of the technical Twitter features (tweets using hashtags are much more likely to receive engagement, while those that mention users are less likely).

**Table 5:** Logit models on receiving engagement and replies.

		**Receiving engagement**				**Receiving replies**			
		**Model 1.1.1**	**Model 1.1.2**	**Model 1.2.1**	**Model 1.2.2**	**Model 2.1.1**	**Model 2.1.2**	**Model 2.2.1**	**Model 2.2.2**
		**Excluding before project observations**	**Excluding before project observations**	**All observations**	**All observations**	**Excluding before project observations**	**Excluding before project observations**	**All observations**	**All observations**
Project phase (Ref.: first phase)								
Bef. project				−1.605^***^ (0.247)	−1.519^***^ (0.191)			0.344 (0.319)	0.548+ (0.314)
Second phase		0.281^*^; (0.114)	0.403^***^ (0.082)	0.281^*^ (0.114)	0.399^***^ (0.087)	−0.600^*^ (0.295)	−0.804^*^ (0.338)	−0.588 (0.299) ^*^	-0.803^*^ (0.332)
After project		0.269+ (0.144)	0.622^***^ (0.099)	0.269+ (0.144)	0.583^***^ (0.083)	−0.668^*^ (0.291)	−0.735^**^ (0.255)	−0.690 (0.291) ^*^	-0.812^**^ (0.255)
Mentioned *chukua hatua*		0.947^*^ (0.373)	0.478 (0.338)	0.947^*^ (0.373)	0.890^**^ (0.318)	−11.681^***^ (0.380)	−12.369^***^ (0.315)	−10.713 (0.377) ^***^	−11.432^***^ (0.307)
Before project x *chukua hatua*				−0.170 (0.394)	−0.293 (0.385)			−0.340 (0.499)	−0.582 (0.440)
Second phase x *chukua hatua*		0.902^***^ (0.265)	0.447 (0.331)	0.902^***^ (0.265)	0.530+ (0.306)	0.573^*^ (0.280)	0.374 (0.380)	0.587^*^ (0.283)	0.341 (0.349)
After project x *chukua hatua*		−0.366 (0.437)	−1.264^*^ (0.601)	−0.366 (0.437)	−1.218^*^ (0.528)	0.663^*^ (0.311)	0.116 (0.396)	0.690^*^ (0.307)	0.161 (0.409)
Engagement (log)						0.192^***^ (0.031)	0.207^***^ (0.034)	0.008 (0.057)	−0.008 (0.069)
Influencer (Ref.: Animator)			−3.277^***^ (0.757)		−3.556^***^ (0.659)		−2.774^***^ (0.684)		−1.720^*^ (0.713)
Region (Ref.: Kigoma)								
Arusha			−0.405 (0.356)		−0.392 (0.300)		−12.977^***^ (0.647)		−12.161^***^ (0.584)
Geita			−0.655^*^ (0.276)		−0.683^*^ (0.266)		−1.143 (1.097)		-1.421 (1.086)
Mtwara			0.562+ (0.336)		0.431 (0.310)		0.197 (0.513)		0.112 (0.473)
Used hashtags			2.118^***^ (0.178)		1.397^***^ (0.177)		−0.750^***^ (0.221)		−0.559^**^ (0.205)
Mentioned users			−0.828^***^ (0.123)		−0.737^***^ (0.117)		−0.090 (0.219)		1.223^***^ (0.305)
Follower count			0.567^***^ (0.118)		0.605^***^ (0.101)		0.188 (0.160)		0.104 (0.164)
Following count			−0.057 (0.115)		−0.082 (0.069)		0.248+ (0.150)		0.064 (0.142)
Tweet count (log)			−0.171 (0.122)		−0.232+ (0.125)		0.029 (0.202)		0.431+ (0.229)
Intercept		0.340 (0.297)	0.394 (1.667)	0.340 (0.297)	1.292 (1.561)	−8.275^***^ (0.344)	−9.689^***^ (2.733)	−7.868^***^ (0.301)	−14.506^***^ (3.030)
Log Likelihood		−281,978.60	−244,819.50	−54,9038.59	−485,484.62	−2,214.06	−2,167.52	−5,287.43	−5,113.42
AIC		563,969.20	489,669.00	1,098,093.18	97,1003.24	4,442.12	4,367.04	10,592.86	10,262.84
BIC		564,035.05	489,833.60	1,098,187.19	971,203.01	4,524.41	4,555.13	10,703.45	10,484.02
Num. obs.		430,866	430,866	937,645	937,645	941,764	941,764	1,603,717	1,603,717

Cluster robust standard errors in parentheses; + p < 0.10,

* p < 0.05,

** p < 0.01,

*** p < 0.001.

Turning to the second set of models focusing on replies, we find a different effect of time: Officials were much less likely to reply to tweets in the later phase of and after the project compared to the first phase. Further, while mentioning *chukua hatua* positively influences the probability to receive engagement by the public, it has a very strong negative effect when explaining replying behavior. Officials were much less likely to reply to tweets using the hashtag and this only varied slightly over time as the interaction effects suggest; compared to the first phase of the project, officials were still less likely to tweets mentioning the term *chukua hatua* and to those not including the term, but a little less so. When focusing on tweets sent during the project time, we find that those which received more engagement are more likely to receive a reply from an official (models 2.1.1 and 2.1.2). Again, the control variables suggest regional variations as well as differences between animators and influencers in the probability to receive replies, and an effect of tweet-specific features.

While the models in Table 5 suggest that officials are more likely to reply to tweets that have received higher levels of engagement, they also show that mentioning *chukua hatua* is negatively affecting a tweet's probability of receiving a reply while it increases its probability to receive some level of engagement in the form of a like or retweet. These findings seem conflicting at first sight, but it might well be that tweets mentioning *chukua hatua* are more likely to be at least liked or retweeted once (potentially by other project participants) while it is the viral tweets which are replied to by the officials and which might not mention the project name.

The models on replying behavior of officials also suggest that tweets are less likely to receive replies since the end of the project. Looking at the data in more detail, we find that officials still regularly reply to influencers and that the last occurrence where an animator received a reply from an official dates back to August 2019, when an animator was in a conversation with an official. In the next section, we will look in more detail at the tweets generating replies and high levels of engagement to gain a better in-depth understanding of the data analyzed.

### Close-reading of big data

The previous sections have shown potential directions on how to analyze large numbers of tweets using quantitative and automatic—distant—methods of data (and text) analysis. The tweets written and published as part of the project do not only need to be analyzed in such a distant way, but much can be gained from a close-reading and in-depth analysis of single tweets and actors. The quantitative analysis can be the starting point for this endeavor.

Tweets written by animators which generated high levels of engagement (over 100 likes/retweets) were, for example, concerned with the issue of water, and read: “#ChukuaHatua “Water is life” is a statement that was made by our leaders in the eighties. However, to date most citizens, especially in our region Geita in Mbogwe district, have water scarcity. The World Bank did a survey at Masumbwe but water availability is still a challenge. What does the ministry say with regard to that. @[mention of account]” (original in Swahili, own translation). Another often retweeted and liked tweet from Arusha featured an image of a school building that fell into a state of disrepair and called for attention, while a popular tweet from Mtwara was concerned with unfavorable feedback received at a village meeting. Looking into the tweets that have generated replies, we, for example, find a tweet written by an animator who also highlights water being an issue in a specific village and who is asking for help. A member of parliament then asked to clarify where the village is located; from the digital trace data, however, we do not know how this continued and whether any action was taken. In another tweet, a minister is being thanked as destroyed bridges and roads are becoming unblocked, and he replies with a positive message for the future. Qualitative evidence can come in to highlight the effectiveness of Twitter in actual cases. In one of the interviews conducted for the study on which this paper builds, an animator remarked: “We tweeted about the shortage of teachers in primary schools and in < 3 months, three teachers were posted”. This was not the only incident, as another animator also mentioned that: “We managed to tweet about the land conflict that occurred at our village then the leaders from the districts came to rescue the situation” (Pretari et al., 2019, p. 51–52). Relying on this interview information, we can state that the project has resulted in real-life offline impact even though it cannot be directly seen from quantitative online evidence. While quantitative analysis of digital trace data has been useful to create a greater overall picture, more in-depth insights can be gained by a close-reading of the tweets produced and in combination with qualitative data sources.

### Conclusion

The Oxfam-led “Governance and Accountability through Digitalization” project in Tanzania has integrated digital technologies into traditional animation approaches, in collaboration with three Tanzanian regional organizations. We have analyzed the content created on Twitter and how the public reacted to it. The analyses have shown that animators signed up to Twitter and started to post on the platform about project-related issues. Additional workshops during the project timeline do not seem to have had an effect on tweeting activity or on engagement received. Animators and influencers have started conversations with key stakeholders, but results suggest that influencers and especially animators only rarely received replies to their tweets. Tweets were more likely to receive replies if they also received high levels of engagement. While explicitly referencing the project in a tweet increased the probability to receive at least one like or retweet, it decreased the probability to receive a reply from a key stakeholder. Even though the overall effect of the project seems to be small according to the analyses discussed—while tweeting activity takes off with the project, reply networks are small in scope, and since the intervention ended, activity is minimal—raising issues on Twitter has shown to lead to positive changes. Nevertheless, despite the positive examples of real-life offline impact mentioned in the previous section, it needs to be acknowledged that the digital component of the project alone did not seem to result in the creation of “large amounts” of bridging social capital, effective network governance and changes in power dynamics considering the relatively low level of engagement of authorities with citizen.

The usage of Twitter data in our study on governance in Tanzania allowed an additional perspective on a developmental project. It has shown to be a valuable complement to the more traditional qualitative and quantitative approaches, specifically allowing to time- and cost-effectively obtain an impression of the potential long-term effects of the project. This has provided us with a unique opportunity to assess the sustainability of the project. While animators have started being active Twitter users, most have stopped tweeting since 2020. However, there is still an exchange of tweets between key stakeholders and influencers, as well as some animators. Our case study also has a number of further limitations and challenges. It is important to keep in mind that we only analyze Twitter data; this does not capture activity on all online channels as WhatsApp is another popular digital tool specifically in Arusha at the regional and district level. Further, while we compare four different regions, it is important to remember their specific dynamics and contexts. These differences can hinder the valid comparison between regions. Also, our analyses do not consider accounts that have been deleted or deactivated from Twitter. This means, we do not know whether animators and influencers have been in active conversation with an official account which has been deleted since. We also tend to underestimate the general level of interaction between animators/influencers and officials by only focusing on replies.

Notwithstanding its limitations, our case study functions as a valuable example highlighting the potential of big data in development sociology. While more in-depth analysis can shed further light on the interesting patterns and dynamics in the project context, we aimed to showcase a starting point for digital trace data in intervention studies.

## Concluding remarks and recommendations

In this paper, we pointed to the advantages and disadvantages of big data analysis in development research, highlighted examples with a sociological perspective, and provided a more in-depth case study taking place in Tanzania serving as an example application with a value-added of digital technologies. Clearly, big data in its various forms can help to shed more light on development issues and there are innovative approaches to measure and predict important indicators related to poverty, social inequality, etc. Solving such measurement problems is certainly an important contribution to development research and practice. However, regarding analyzing and explaining the effectiveness of development programs and interventions, a key aim of development initiatives and research, the contribution of big data is less straightforward. The reason is that quantitative impact evaluations rely on causal inference built on counterfactual logic, operationalized through treatment and control group(s), and this is not a given if big data are “just” used as an additional data source. In other words: To provide useful insights regarding the effectiveness of interventions, big and digital trace data need to be considered in the research design phase of development research. Our example has limitations regarding such a causal analysis (e.g., regions in our study do not only vary regarding the social media intervention but also other characteristics). Yet it also demonstrates how a causal analysis can be implemented as part of an impact evaluation. Furthermore, our study exemplifies one major advantage of using (big) digital data as part of an intervention study: Digital data allow the study of long-term effects of interventions which is certainly a limitation in most intervention studies. In our case study, social media activity is significantly decreasing in the long term. Yet, this picture would have looked quite different when only considering the actual time when the intervention project was running.

At first sight, our case study might suggest a limited effect of social media on governance in the corresponding regions in Tanzania. However, we also find some sustained social media activity as well as testimonies of online activity as part of the intervention sparking actual changes at the community level. This underscores the importance of combining different data sources and strategies of analysis in development research. Quantitative analysis of big data is not meant to replace other approaches but to complement them, and there is a need for cross-validation. From a sociological perspective, it is furthermore evident that phenomena such as citizenship and governance are complex and multifaceted and hence their study demands a comprehensive research design, combining qualitative and quantitative research components. In fact, our social media case study was part of a much larger evaluation that comprised several components (see Pretari et al., 2019). Sociology also invites us to reflect on who is considered a knowledge producer and to be attentive to the power imbalances between different epistemologies. We discuss below the potential for big data research to promote a different role for data producers in the research process and provide a table of recommendations (see Table 6).

**Table 6:** Issues/challenges of big data analysis in the context of development sociology and recommendations.

**Issues/challenges**	**Recommendations**
*Uncovering causal effects*: A control-treatment group design is not inherent to big data.	An experimental setup should be considered in the planning phase of development research.
*Estimating long-term effects*: Treatment effects often fade away briefly after the intervention which is difficult to measure.	The advantage of big data to be more easily collected repeatedly should be used. This can shed light on the persistence of intervention effects.
*Evaluating the impact*: Big data might fall short of capturing all aspects of impact.	Typically, big data analysis is one part of a (complex) development research project and should be complemented with other forms of qualitative and/or quantitative data analysis.
*Deriving representative conclusions*: Big data such as social media data might be based on biased samples.	Researchers should acknowledge and try to estimate a potential sample bias of big data such as social media data.
*Collecting ethically sound data*: Typically, big data is not produced for research purposes.	Whenever possible, researchers need to obtain informed consent from research participants such as social media users. In any case, they need to consider ethical aspects such as respect for persons, beneficence, justice, and respect for law and public interest.
*Engaging people who produced the (big) data*: There is a danger that research participants have no say in the research process, as well as of power and racial dynamics to be reproduced in the way knowledge is generated.	Research teams should find ways for the data producers to become the researchers of the data they produce and participate in the research process for example by a strong community engagement.
*Providing public access to data*: Many studies using big data cannot be replicated due to lack of access to data and code.	In line with general developments toward open data in development research, researchers at both universities and NGOs should strive for making data and code publicly available (while considering ethical and legal restrictions).

There are various important aspects that need to be addressed in big data analysis. Particularly, the representativeness and ethical aspects of this type of analysis must be discussed (Lazer et al., 2014; Ruths and Pfeffer, 2014; Townsend and Wallace, 2016). Representativeness can be affected by potential biases in the available data, but also by the fact that users of social media platforms can differ in their characteristics from the general population. It is important to be aware of digital divides and inequalities within the study population when making general claims. From an ethical standpoint, (big) digital data comes with new questions and uncertainties which are also best addressed and considered in the research design phase. Even if only publicly available information is accessed (as is generally the case with Twitter), it is important to keep in mind that users of social media sites make their data available for the purpose of social networking, not to be harvested and used for research purposes. Salganik (2018, Chapter 6) advises to be guided by four principles when facing ethical uncertainty in digital social science research: respect for persons, beneficence, justice, and respect for law and public interest. Townsend and Wallace (2016) also highlight that the terms of conditions of social media sites, the ethical guidelines of the researcher's institution, the privateness of the social media site, the vulnerability of the users, the sensitivity of the research topic, and the potential for anonymization and data sharing must be considered when making use of digital trace data (see also Zook et al., 2017 for responsible big data research). In the ideal case and whenever possible, researchers should obtain consent of their participants. This is especially indispensable when wanting to access more private digital spaces like WhatsApp groups or similar. The use of big data in sociological research also raises the broader question of how to engage the many people who produced the data in the research process. Going beyond the consent stage, engagement means for data producers to shape the research questions, the analysis, interpretation, and ultimately its use. In the setting of development research, the racial division of labor has been documented [see for a recent example the “(Silent) Voices Bukavu series” blog^5^]. We acknowledge that the team of researchers involved in this research is primarily white, living in and from the so-called Global North. While big data research and computational social science in general promote a culture of open access of codes and availability of data, more effort needs to be devoted to challenge power dynamics and avoid structural inequalities to be reproduced in and through the research field. In particular, big data development sociology as a sector and sociologists as individuals need to challenge racialized power dynamics between research teams and data producers, and find ways for the data producers to become the researchers of the data they produce and participate in the research process.

Our paper aimed at presenting the potential of different types of big data for development sociology and an example case study integrating social media in an intervention program. This can be seen as a starting point for more systematic usage of big data in development research with a sociological focus. It should be clear that this sociological focus comprises a vast number of sociologically relevant topics that can be studied with big data, as well as different techniques such as network or text analysis which can be applied to big data in the context of development research. A sociological focus also entails the integration of a sociological perspective in inter- and transdisciplinary research including a critical reflection on the use of big data in the development sector. We hope that our paper paves the way for much more research on these topics.

## Data availability statement

The raw data supporting the conclusions of this article will be made available by the authors, without undue reservation.

## Ethics statement

For the Oxfam-led project informed consent was obtained from all participants. While no informed consent was obtained for the Twitter data analysis, only publicly available information was collected, i.e. profiles which were made private or deleted after the project were not collected, and deleted tweets were not collected. Further, Twitter data were not merged with other personal data.

## Author contributions

WM (with Oxfam in Tanzania and partner organizations) designed and carried out the project. AP and SL designed and implemented its impact evaluation, in collaboration with WM (and Oxfam in Tanzania and partners). NS, UL, AP, and SL designed the Twitter data analysis. NS collected and analyzed the data. NS and UL prepared the draft manuscript with input from all authors. All authors reviewed the results and approved the final version of the manuscript.

## Funding

The presented case study was funded by the Belgian Directorate-General for Development Cooperation and Humanitarian Aid. UL acknowledges support by the Warwick ESRC Impact Acceleration Account.

## Conflict of interest

Authors AP, WM, and SL were employees of Oxfam at the time of the project.

The remaining authors declare that the research was conducted in the absence of any commercial or financial relationships that could be construed as a potential conflict of interest.

## Publisher's note

All claims expressed in this article are solely those of the authors and do not necessarily represent those of their affiliated organizations, or those of the publisher, the editors and the reviewers. Any product that may be evaluated in this article, or claim that may be made by its manufacturer, is not guaranteed or endorsed by the publisher.

## Footnotes

1 Oxfam is an international confederation of charitable organizations focusing on the alleviation of global poverty, founded in Oxford in 1942.

2 For this previous project, villages were selected by partner organizations based on the relationships they had held already and the trust they had built in the past (see on the former project Smith and Kishekya, 2013; Green, 2015).

3 We also want to note that some discrepancies in numbers reported here and in the previous impact evaluation (Pretari et al., 2019) stem from the fact that only publicly available users and tweets are being collected via the API. If user accounts as a whole or specific tweets are temporarily or permanently deactivated/deleted, this information will not be retrieved. While information collected at different points in time could be merged to achieve a dataset of better quality, we want to respect users' right to be forgotten and their ability to delete, deactivate, or privatize their information previously shared. We thus only use data that is available at the time of data collection (November 2021).

4 It is organized by the Elimika Wikiendi Company LTD (https://www.elimika.co.tz/).

5 A blog series which highlights ‘the premeditated violence that persists in the process of academic knowledge production', arguing ‘that this process is, among other things, responsible for the dehumanization and the erasure of researchers from the Global South, available here https://www.gicnetwork.be/silent-voices-blog-bukavu-series-eng/
