# Supplementary material for: Big data and development sociology: An overview and application on governance and accountability through digitalization in Tanzania
Source: Front Sociol. 2022 Nov 17;7:909458. doi: 10.3389/fsoc.2022.909458 (PMC9712952; doi:10.3389/fsoc.2022.909458)
Supplement: Supplementary file 1 [file Table_1.DOCX]

**Appendix**

**List of Twitter accounts for various Government officials in Tanzania (local and national level), and for institutions**

1. National level leaders (Ministers, Prime Minister, Vice President, President)

| **Name** | **Title/Position** | **Twitter Account / Link** |
| --- | --- | --- |
| John Pombe Magufuli | President of the United Republic of Tanzania | <https://twitter.com/MagufuliJP> |
| Samia Suluhu | Vice President of the United Republic of Tanzania | <https://twitter.com/SuluhuSamia> |
| Vice President's Office | Official Twitter account for office of the Vice President of Tanzania | <https://twitter.com/VPOTanzania> |
| Majaliwa Kassim | Prime Minister of the United Republic of Tanzania | <https://twitter.com/majaliwa_kassim> |
| January Makamba | Minister of State in the Vice President’s Office for Union Affairs and Environment. Member of Parliament for Bumbuli Constituency. | <https://twitter.com/JMakamba> |
| Prof. Makame Mbarawa | Member of Parliament, Minister for Water and Irrigation, Former Minister for Works, Transport and Communications | <https://twitter.com/MbarawaM> |
| Abdallah Hamis Ulega | Ministry of Agriculture | <https://twitter.com/tzagriculture> |
| Angellah Kairuki | Member of Parliament and Minister of Mining | <https://twitter.com/AngellahKairuki> |
| Luhaga Joelson Mpina | A member of Parliament for Kisesa Constituency and a Minister for Livestock and Fisheries | <https://twitter.com/LuhagaM> |
| Prof. Paramagamba John Kabudi | Ministry of Justice and Constitution Affairs | <https://twitter.com/Sheria_Katiba> |
| Dr. Augustine Philip Mahiga | Ministry of Foreign Affairs and International Co-operation | <https://twitter.com/Balozi_Mahiga> |
| Ummy Ally Mwalimu | Minister for Health, Community Devl., Gender, Elderly & Children, Tanzania 2015 to present / Member of Parliament, | <https://twitter.com/umwalimu> |
| Dr. Harrison Mwakyembe | Ministry of Information, Youth, Culture and Sports | <https://twitter.com/Wizara_HabariTZ> |
| Hamis Andrea Kigwangalla | Minister for Natural Resources and Tourism \| Member of Parliament, Nzega Rural \| Philosopher turned a Doct | <https://twitter.com/Hkigwangalla> |
| Prof. Makame Mbarawa Mnyaa | Minister for Water and Irrigation | <https://twitter.com/MbarawaM> |
| Isack Aloyce Kamwelwe | Ministry of works transport and communications | <https://twitter.com/IsackKamwelwe> |
|  | The official account of the Chief Spokesperson of the Government of Tanzania | <https://twitter.com/TZMsemajiMkuu> |
|  | Ministry of Internal Affairs, official spokesman account | <https://twitter.com/WizaraMNN> |

1. Local level leaders (Village, District, Regional, Councillors, Member of parliament)

| **Name** | **Title/Position** | **Twitter Account / Link** |
| --- | --- | --- |
| Juma Mnwele | District Executive Director - Kibondo | <https://twitter.com/JMnwele> |
| Ally Salum Hapi | Regional Commissioner - Iringa | <https://twitter.com/AllyHapi> |
| Paul Makonda | Regional Commissioner - Dar es salaam | <https://twitter.com/Paul_Makonda> |
| Anna Mghwira | Regional Commissioner - Kilimanjaro | <https://twitter.com/AnnaMghwira> |
| Mrisho Gambo | Regional Commissioner - Arusha | <https://twitter.com/mrishogambo> |
| Evarist Ndikilo | Regional Commissioner - Pwani | <https://twitter.com/welendikilo> |
| Aggrey Mwanri | Regional Commissioner - Tabora | <https://twitter.com/AggreyMwanri> |
| John Mongella | Regional Commissioner - Mwanza | <https://twitter.com/john_mongella> |
| Raphael Muhuga | Regional Commissioner - Katavi | <https://twitter.com/raphael_muhuga> |
| David Kafulila | Administrative secretary - Songwe | <https://twitter.com/kafulila_david> |
| Jerry Cornel Muro | District Commissioner - Arumeru | <https://twitter.com/jerrycornelmuro> |
| Jokate Mwegelo | District Commissioner - Kisarawe | <https://twitter.com/jokateM> |
| Sophia Kizigo | District Commissioner - Namtumbo | <https://twitter.com/SophiaMfaume> |

1. Institutions (Relevant to the project)

| **Name** |  | **Account** |
| --- | --- | --- |
| Tanesco | Public Electricity Company in Tanzania | <https://twitter.com/tanescoyetu> |
| TCRA | Tanzania Communications Regulatory Authority | <https://twitter.com/TCRA_Tz> |
| TPA | Tanzania Ports Authority | <https://twitter.com/tanzaniaportshq> |
| PCCB | Prevention and Combating of Corruption Bureau | <https://twitter.com/takukuru_tz> |
| SUMATRA | Surface and Marine Transport Regulatory Authority | <https://twitter.com/SumatraTZ> |
| TIC | Tanzania Investment Centre | <https://twitter.com/InvestinTanzani> |
| TFS | Tanzania Forest Service | <https://twitter.com/tfs_tz?lang=en> |
| BRELA | Business Registrations and Licensing Agency | <https://twitter.com/BRELA_Tz?lang=en> |
| BOT | Bank Of Tanzania | <https://twitter.com/BankOfTanzania?lang=en> |
| TIRA | Tanzania Insurance Regulatory Authority | <https://twitter.com/TIRA_TZ?lang=en> |
| TFDA | Tanzania and Drugs Authority | <https://twitter.com/tfdatanzania?lang=en> |
| TASAF | Tanzania Social Action Fund | <https://twitter.com/TASAFIII?lang=en> |
| EPZA | Export Processing Zone Authority | <https://twitter.com/EPZA_Tanzania?lang=en> |
| NIDA | National Identification Authority | <https://twitter.com/NIDA_Tanzania> |
| NHIF | National Health Insurance Fund | <https://twitter.com/nhiftz> |
| Cashewnut Board of Tanzania | Cashewnut Board of Tanzania | <https://twitter.com/CashewTanzania> |
| Oxfam | Oxfam in Tanzania | <https://twitter.com/OxfamTz> |
| LHRC | Legal and Human Rights Center | <https://twitter.com/humanrightstz> |
| HakiElimu | HakiElimu | <https://twitter.com/HakiElimu> |
| Policy Forum | Policy Forum | <https://twitter.com/policy_F> |
| Haki Rasilimali | Haki Rasilimali | <https://twitter.com/HakiRasilimali> |
| Chukua Hatua | Chukua Hatua | <https://twitter.com/ChukuaHatua> |
| CABUIPA | Capacity Building Initiative for Poverty Alleviation | <https://twitter.com/cabuipa> |
| TWAWEZA | TWAWEZA ni sisi | <https://twitter.com/Twaweza_NiSisi> |
| MSOAPO | Mtwara Society Against Poverty | <https://twitter.com/msoapomtwara> |
